# Supplementary material for: High-Molecular-Weight Exopolysaccharides Production from Tuber brochii Cultivated by Submerged Fermentation
Source: Int J Mol Sci. 2023 Mar 2;24(5):4875. doi: 10.3390/ijms24054875 (PMC10002917; doi:10.3390/ijms24054875)

Table S1. Effect of different yeast extract concentrations on mycelia growth and polysaccharides production from *T. borchii.*

Yeast extract concentration (g/L)	Dry cell weight (g/L)	EPS (mg/L)	IPS (mg/L)
10	3.41±0.04	498.74±13.24	929.44±12.36
15	3.54±0.21	603.6±28.14	1064.75±96
20	4.06±0.11	725.2±30.17	1385.7±41.03
25	1.65±0.29	776.09±29.72	667.39±34.38
30	1.27±0.22	905.61±4.85	411.07±103.05

Table S2. Strain Identification Report

**Committee Support Unit**	Middle Institute of Chemical Engineering, Hsingh University	**Receive Inspection Date**	2021/04/20
**Committee Trustee**	Liu Yong Quan	**Report Announcement date**	2021/05/03
**Check body type**	■Tray□Bacteria □Bacteria powder □Other, please specify:		
**Check test items**	□Identification of bacterial strains ■Identification of fungal strains		
**Check body name**	*Tuber borchii* (ATCC-MYA-1019)		
**Remark**			

Detection process description:

DNA extraction → Target PCR → Sequential decoding → Sequence Alignment → Evolutionary tree → Report

DNA Extraction: Taco automatic nucleic acid extractor (magnetic bead system) was used to extract and purify the DNA of bacteria or fungi. For detailed procedures, please refer to Total User Manual for DNA Extraction Kit (product code: atci-dna).

Target PCR-1: 16S rDNA is used as the target gene for bacterial identification. Considering that the diversity of bacterial species makes PCR difficult to amplify, three different sets of Universal Primers were used for 16S rDNA PCR amplification, and the PCR products that were successfully amplified and longer in length were sequenced and decoded. The PCR product sizes of these three sets of primers are listed in the table below:

Numbering	Primer name	PCR product (bp)	References
01	27F/1525R	1500	Lane, DJ 1991. 16S/23S rRNA sequencing. In: Nucleic acid techniques in bacterial systematics. Stackebrandt, E., and Goodfellow, M.,eds., John Wiley and Sons, New York, NY, pp. 115-175
02	8F2/806R	802	N Engl J Med 1992;327:293-301
03	fD1modF/16S1RR-B	568	J Clin Microbiol 1998;36:2205-9.<br>Arthritis Rheum 1998;41:535-43.

Target PCR-2: Fungal identification uses NS (18S) or ITS (18S/28S) as the target gene. Considering that the diversity of fungal species makes PCR difficult to amplify, 3 different sets of Universal Primers were used for NS or ITS PCR amplification, and the PCR products that were successfully amplified and longer in length were sequenced and decoded. The PCR product sizes of these three sets of primers are listed in the table below:

Numbering	Primer name	PCR product (bp)	References
01	NS1/NS6	1400	White TJ, T. Bruns, S. Lee and J. Taylor, in PCR Protocols: A Guide To Methods And Applications, ed. MA Innis, DH Gelfand, JJ Sninsky, TJ White, Academic Press, San Diego, 1990, pp. 315–322.
02	NS5/NS6	310	
03	ITS1/ITS4	570/590	

PCR conditions and reaction temperature:

Reaction mixture		Reaction time	Instrument
**Reaction components**	**Volume (μL)**		Veriti 96 Well Thermal Cycler<br>Serial Number:#299025707
• Ultrapure water	8.4	95 °C- 5 min	
• 2.5mM dNTP	3.2	95 °C- 30 sec (40- Cycles)	
• 10X reaction buffer	2	60 °C- 30 sec (40- Cycles)	
• 25mM $MgCl_2$	1.2	72 °C- 30 sec (40- Cycles)	
• 100% DMSO	1	72 °C- 7 min	
• SuperTherm Taq Polymerase 1U/μL	0.2	Stop	
• 2μM F+R primers	2		
• DNA template	2		
• Total	20		

Test result:

Nucleic acid sequencing results: Tuberaceae (taxid:40289)

TTGCTATCGTCCYTYTGCCCTATCGGACTCCCAAGCAAAACACATTCCTGTG TACTCTCCCTCATTAAACTTTTGAACCAATTAGTAGTCTGAGAAGGC

CATGTGCCGTAAAATTTAAACATGTTAAAACTTTCAACAACGGATCTCTTGG CTCTCGCATCGATGAAGAACGCAGCGAAATGCGATAAGTAATGTGAA

TTGCAGAATTCAGTGAATCATCGAATCTTTGAACGCACATTGCGCCCTTTGG TATTCCTTAGGGCATGCCTGTTCGAGCGTCACTAAAAACCCCCTCAC

AGATAATGTGGTATTGGTAGAAGTGGATGGTACTAACACTACTTTGTCTGAC TCTACTGAAATGAATAGGCCAGAGAAGTTGACCGTGGTAATAGACTC CAGGAGTGTTTTTAAAATGCTAAATTAGTCTTCTCCAAGTCATGTTCTGAGC TACCGGACCCCCCATTTAGTCCAAACTAGAGAGGTTGACCTCGGATCAGGT AGGGATACCCGCTGAAAGCGATAGAATCA

NuclearAcid sequence alignment results:

Sequence column comparisons takes into account 5 parameters, as described below

Max Score: The highest alignment score from that database sequence

Total Score: The total alignment scores from all alignment segments

Query Cover: Query covered by alignment to the database sequence

E value: The best (lowest) expect value of all alignments from that database sequence

Identify: The highest percent identity (Max ident of all query-subject alignments)

Both Max Score and Total Score values indicate the homology of the two sequences, and the higher the value, the greater the degree of similarity between them.

E value value is used to evaluate Max Score and Total Score numbers value reliability. It means that in a random situation, the similarity between other sequences and the target sequence is greater than Max Score and Total Score numbers possibility of value. Therefore, the lower the value of E value, the higher the reliability.

Knot Conclusion: The results of NCBI Blast comparison show that the sample has the highest similarity with the following strains:

1. ***Tuber* sp. MS11 18S ribosomal RNA gene, partial sequence; internal transcribed spacer 1, 5.8S ribosomal RNA gene, and internal transcribed spacer 2, complete sequence; and 28S ribosomal RNA gene, partial sequence Sequence ID: KF850621.1**

	Description	Max Score	Total Score	Query Cover	E value	Per. Ident	Accession
☑	Tuber sp. MS11 18S ribosomal RNA gene, partial sequence; internal transcribed spacer 1, 5.8S ribosomal RNA gene, and internal transcribed spacer 2, complete sequence; and 28S ribosomal RNA	970	970	97%	0.0	100.00%	KF850621.1
☐	Tuber borchii 17S ribosomal RNA, partial sequence; internal transcribed spacer 1, 5.8S ribosomal RNA, and internal transcribed spacer 2, complete sequence; and 25S ribosomal RNA, partial seque	968	968	97%	0.0	100.00%	AF250291.1
☐	Tuber borchii isolate GB62 18S ribosomal RNA gene, partial sequence; internal transcribed spacer 1, 5.8S ribosomal RNA gene, and internal transcribed spacer 2, complete sequence; and 28S ribo	966	966	97%	0.0	99.81%	HM485342.1
☐	Tuber borchii voucher GB32 collection-date 05-Feb-2007 internal transcribed spacer 1, partial sequence; 5.8S ribosomal RNA gene and internal transcribed spacer 2, complete sequence; and 28S la	965	965	97%	0.0	99.81%	FJ809852.1
☐	Tuber borchii strain IG2 internal transcribed spacer 1, partial sequence; 5.8S ribosomal RNA gene and internal transcribed spacer 2, complete sequence; and 28S ribosomal RNA gene, partial seque	963	963	97%	0.0	99.81%	KF414978.1
☐	Tuber borchii isolate GB39 internal transcribed spacer 1, partial sequence; 5.8S ribosomal RNA gene and internal transcribed spacer 2, complete sequence; and 28S ribosomal RNA gene, partial se	959	959	97%	0.0	99.62%	HM485343.1
☐	Tuber borchii voucher Tar202 internal transcribed spacer 1, partial sequence; 5.8S ribosomal RNA gene and internal transcribed spacer 2, complete sequence; and 28S ribosomal RNA gene, partial	953	953	95%	0.0	100.00%	KT165341.1
☐	Tuber borchii voucher Tar147 18S ribosomal RNA gene, partial sequence; internal transcribed spacer 1, 5.8S ribosomal RNA gene, and internal transcribed spacer 2, complete sequence; and 28S ri	953	953	95%	0.0	100.00%	KT165333.1
☐	Tuber borchii voucher Tar136 18S ribosomal RNA gene, partial sequence; internal transcribed spacer 1, 5.8S ribosomal RNA gene, and internal transcribed spacer 2, complete sequence; and 28S ri	953	953	95%	0.0	100.00%	KT165330.1
☐	Tuber borchii strain ATCC 96540 18S ribosomal RNA gene, partial sequence; internal transcribed spacer 1, 5.8S ribosomal RNA gene, and internal transcribed spacer 2, complete sequence; and 28	953	953	95%	0.0	100.00%	HQ026725.1

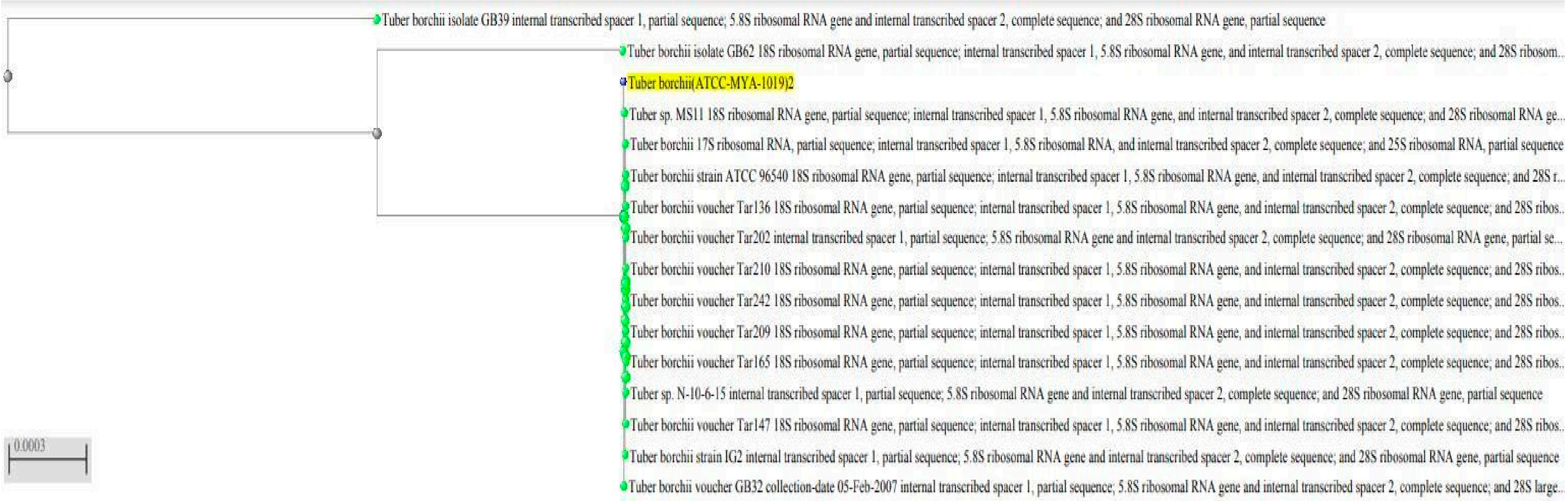
Tuber borchii isolate GB39 internal transcribed spacer 1, partial sequence; 5.8S ribosomal RNA gene and internal transcribed spacer 2, complete sequence; and 28S ribosomal RNA gene, partial sequence
Tuber borchii isolate GB62 18S ribosomal RNA gene, partial sequence; internal transcribed spacer 1, 5.8S ribosomal RNA gene, and internal transcribed spacer 2, complete sequence; and 28S ribosom...
Tuber borchii(ATCC-MYA-1019)2
Tuber sp. MS11 18S ribosomal RNA gene, partial sequence; internal transcribed spacer 1, 5.8S ribosomal RNA gene, and internal transcribed spacer 2, complete sequence; and 28S ribosomal RNA ge...
Tuber borchii 17S ribosomal RNA, partial sequence; internal transcribed spacer 1, 5.8S ribosomal RNA, and internal transcribed spacer 2, complete sequence; and 25S ribosomal RNA, partial sequence
Tuber borchii strain ATCC 96540 18S ribosomal RNA gene, partial sequence; internal transcribed spacer 1, 5.8S ribosomal RNA gene, and internal transcribed spacer 2, complete sequence; and 28S r...
Tuber borchii voucher Tar136 18S ribosomal RNA gene, partial sequence; internal transcribed spacer 1, 5.8S ribosomal RNA gene, and internal transcribed spacer 2, complete sequence; and 28S ribos...
Tuber borchii voucher Tar202 internal transcribed spacer 1, partial sequence; 5.8S ribosomal RNA gene and internal transcribed spacer 2, complete sequence; and 28S ribosomal RNA gene, partial se...
Tuber borchii voucher Tar210 18S ribosomal RNA gene, partial sequence; internal transcribed spacer 1, 5.8S ribosomal RNA gene, and internal transcribed spacer 2, complete sequence; and 28S ribos...
Tuber borchii voucher Tar242 18S ribosomal RNA gene, partial sequence; internal transcribed spacer 1, 5.8S ribosomal RNA gene, and internal transcribed spacer 2, complete sequence; and 28S ribos...
Tuber borchii voucher Tar209 18S ribosomal RNA gene, partial sequence; internal transcribed spacer 1, 5.8S ribosomal RNA gene, and internal transcribed spacer 2, complete sequence; and 28S ribos...
Tuber borchii voucher Tar165 18S ribosomal RNA gene, partial sequence; internal transcribed spacer 1, 5.8S ribosomal RNA gene, and internal transcribed spacer 2, complete sequence; and 28S ribos...
Tuber sp. N-10-6-15 internal transcribed spacer 1, partial sequence; 5.8S ribosomal RNA gene and internal transcribed spacer 2, complete sequence; and 28S ribosomal RNA gene, partial sequence
Tuber borchii voucher Tar147 18S ribosomal RNA gene, partial sequence; internal transcribed spacer 1, 5.8S ribosomal RNA gene, and internal transcribed spacer 2, complete sequence; and 28S ribos...
Tuber borchii strain IG2 internal transcribed spacer 1, partial sequence; 5.8S ribosomal RNA gene and internal transcribed spacer 2, complete sequence; and 28S ribosomal RNA gene, partial sequence
Tuber borchii voucher GB32 collection-date 05-Feb-2007 internal transcribed spacer 1, partial sequence; 5.8S ribosomal RNA gene and internal transcribed spacer 2, complete sequence; and 28S large...
0.0003

S/N G:92 A:86 T:43 C:54

KB.bcp

KB 1.4.1.8 Cap:28

ITS1-ITS4-Tuber borchii(ATCC-MYA-1019)2_ITS1

ITS1-ITS4-Tuber borchii(ATCC-MYA-1019)2_ITS1

KB_3730_POP7_BDTv3.mob

Pts 1763 to 9216 Pk1 Loc:1762

Version 5.2.0 HiSQV Bases: 512

Inst Model/Name 3730/NT1413-1413-012

April 27,2021 03:32 a.m., GMT

April 27,2021 03:55 a.m., GMT

Spacing:14.67 Pts/Panel1500

Plate Name: 210426M1

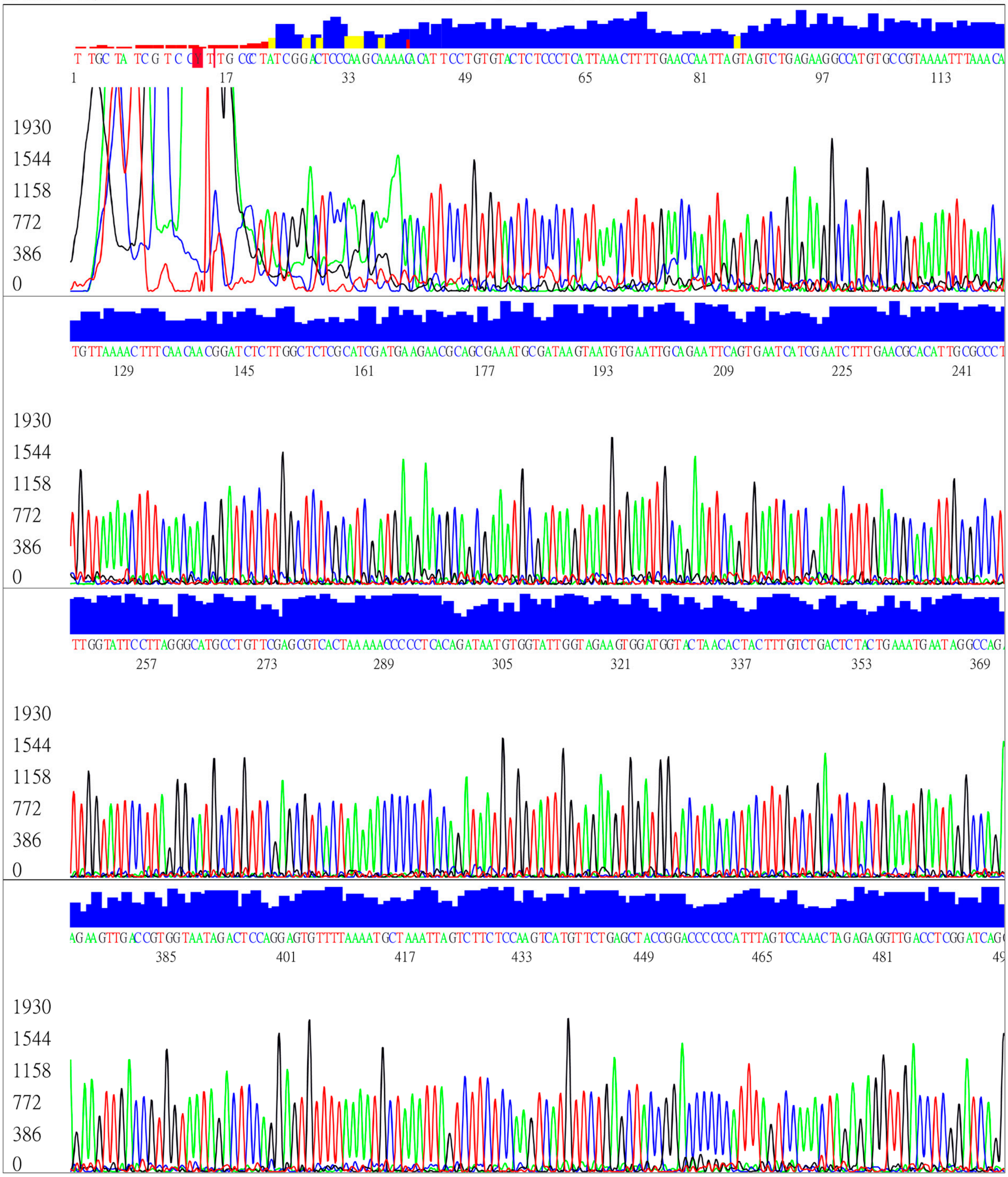

Printed on: Wednesday April 28,2021 03:11 a.m., GMT

Electropherogram Data Page 1 of 2

Applied Biosystems

ITS1-ITS4-Tuber borchii(ATCC-MYA-1019)2_ITS1
ITS1-ITS4-Tuber borchii(ATCC-MYA-1019)2_ITS1

Inst Model/Name 3730/NT1413-1413-012
April 27,2021 03:32 a.m., GMT
April 27,2021 03:55 a.m., GMT

S/N G:92 A:86 T:43 C:54
KB.bcp
KB 1.4.1.8 Cap:28

KB_3730_POP7_BDTv3.mob
Pts 1763 to 9216 Pk1 Loc:1762
Version 5.2.0 HiSQV Bases: 512

Spacing:14.67 Pts/Panel1500
Plate Name: 210426M1

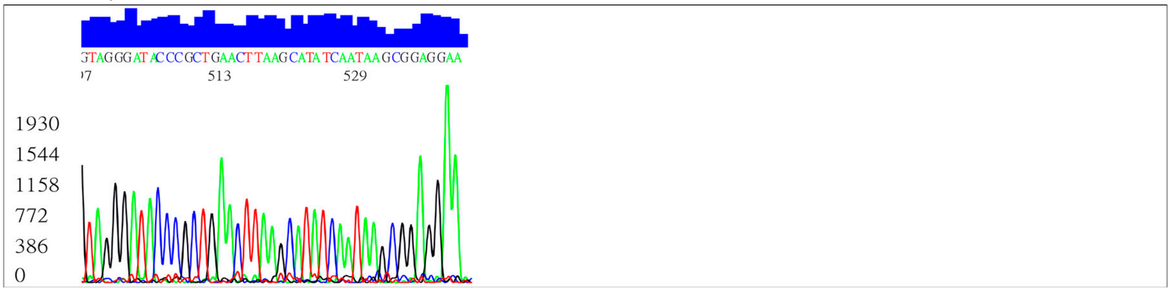

Supplement: Supplementary file 1 [file ijms-24-04875-s001.zip › ijms-2197382-supplementary.pdf]
